# Robust regulation of a PVTOL aircraft subject to crosswind disturbances: Passivity and integral sliding mode approach

**DOI:** 10.1371/journal.pone.0307398

**Published:** 2024-12-05

**Authors:** Carlos Aguilar-Ibanez, Miguel S. Suarez-Castanon, Belem Saldivar, Manuel Jiménez-Lizárraga, de Jesus Jose Rubio

**Affiliations:** 1 Centro de Investigación en Computación, Instituto Politécnico Nacional, Ciudad de México, Mexico; 2 Escuela Superior de Cómputo, Instituto Politécnico Nacional, Ciudad de México, Mexico; 3 Departamento de Control Automático, Centro de Investigación y de Estudios Avanzados del Instituto Politécnico Nacional, Ciudad de México, Mexico; 4 Facultad de Ciencias Físico Matemáticas, Universidad Autónoma de Nuevo León, Mexico; 5 Escuela Superior de Ingeniería Mecánica y Eléctrica, Instituto Politécnico Nacional, Ciudad de México, Mexico; University of Shanghai for Science and Technology, CHINA

## Abstract

This paper deals with the problem of robust regulation of a Planar Vertical Takeoff and Landing (PVTOL) aircraft affected by crosswind disturbances. The proposed approach simultaneously exploits the advantages of passivity-based control and the integral sliding mode (ISM) control technique. We demonstrate that the passivity-based controller accomplishes the PVTOL regulation within a guaranteed stability region. At the same time, the ISM controller can effectively counteract the undesirable disturbances effect from the beginning of the operation since the disturbances are never seen in the measurable output. Using the passivity approach with LaSalle’s Invariance Theorem, we construct the required Lyapunov function to establish the closed-loop system stability. The proposed combination of controllers results in a robust system that satisfactorily solves the regulation problem. Numerical simulations are provided to show that the proposed approach allows the tracking of different reference signals: pulse-train shaped trajectory and ellipse-shaped trajectory, considering the presence of perturbations (crosswind). Furthermore, to illustrate the effectiveness of the proposed technique, a comparative analysis considering two robust control strategies is provided.

## 1 Introduction

Due to the complex nonlinear dynamics of unmanned aerial vehicles, designing control techniques to make them accomplish even simple tasks like reaching a point and staying stable at it has proven to be very challenging. Fortunately, the simplified unmanned Partial Vertical Take Off and Landing (PVTOL) aircraft introduced more than forty years ago embodies several characteristics and behaviors of aircrafts like quadrotors, helicopters or the Harrier Jump Jet [1, 2]. Consequently, this aircraft has been used as a testbed for existing control techniques and the design of new ones. Additionally, due to its simplicity, it is relatively easy and inexpensive to implement this system to perform experiments, allowing for adjustments to the controller, if needed, and then implement it in actual applications.

Some contributions that address the control design for a PVTOL aircfraft are briefly described below. In [3], Hauser *et. al.* designed a stabilizing controller by using the feedback linearization technique for a non-minimum phase approximation of the PVTOL system. It was shown that the direct application of the exact input-output linearization technique to the flight control of vertical take-off and landing aircraft is not effective. The proposed technique works only if the coupling between the rolling moment input to the aircraft dynamics and the dynamics along the y-axis are decoupled. Furthermore, this approach does not consider the presence of external disturbances.

The result presented in [3] was outperformed by the proposal presented in [4], where Martin *et al.* studied the non-minimum phase outputs regulation of the PVTOL system. The flatness property of this aircraft was exploited to use the inverse trajectory feedforward and a state tracker to ensure bounded zero dynamics. Numerical simulations confirm the superiority of the proposed method with respect to the proposal presented in [3], however, it does not consider the presence of disturbances either.

Inspired by the results presented in [4], Sira-Ramirez and Fliess proposed in [5, 6] a solution for the trajectory tracking problem for the PVTOL. The proposed approach, which also exploits the flatness property of the system, differs from the one presented in [4], in the proposed differential parametrization which is naturally provided by the flatness property. This approach allows establishing the PVTOL center of gravity coordinates in terms of the minimum phase flat output vector coordinates. The required dynamical feedback controller is then obtained by solving a suitable trajectory tracking problem, with linear tracking error dynamics. Numerical simulations confirm the effectiveness of the proposed control approach.

Based on the forwarding control approach, an algorithm that ensures asymptotic stability using a Lyapunov function was presented by Fantoni and Lozano in [7]. The proposed control approach assumes that there is no coupling between rolling moments and lateral acceleration. Then, the authors expect to get a loss of performance due to the unmodeled dynamics present in the system. Numerical simulations show a slow convergence of the system state to the desired reference signal.

Inspired by the results presented in [7], similar solutions for the PVTOL actual applications were presented in [8–12]. In [11], using nonlinear combinations of saturation functions bounding the thrust input to arbitrary saturation limits, Zavala-Río *et al.* designed a globally stabilizing control for a PVTOL aircraft. As in [7], the proposed control approach assumes that there is no coupling between the rolling moment and the lateral acceleration of the aircraft. To validate the proposal, numerical simulations are reported. Saturation bounds on the controllers are assumed, however, as in [7], a slow convergence of the state is observed. For comparison purposes, the case where the controllers are unbounded is considered, a reduction of the response time is obtained but large values of the control inputs are observed.

In [13], using Robust Control Lyapunov Functions and Sontag’s universal stabilizing feedback, Muñoz *et al.* address the problem of robust stabilization of a PVTOL aircraft in the presence of crosswind. Numerical simulation and experimental results illustrate the effectiveness of the proposal. Although displacements of the PVTOL aircraft position can be observed, low-cost inputs are achieved.

An algorithm based on a robust control Lyapunov function approach and unknown disturbances rejection for stabilizing a wind-perturbed vertical take-off and landing aircraft is introduced in [14]. To evaluate the viability of the control scheme in real-time application a PVTOL platform prototype is employed. As in [13], even though state displacement is observed, the PVTOL remains stable with low-cost inputs.

A novel exact linearization procedure based on the implicit systems techniques is proposed by Bonilla *et al.* in [15]. This technique divides the nonlinear state representation into a basic rectangular representation and an auxiliary nonlinear algebraic equation. The proposed control design methodology can be applied to sophisticated nonlinear dynamic systems. The applicability of the proposed implicit systems description based control design is illustrated by solving the regulation problem for the PVTOL system. Numerical simulations show satisfactory outcomes.

A robust structural feedback linearization technique is proposed by Bonilla *et al.* in [16]. The proposed approach is relatively simple and involves a generic linear-type control scheme based on the classic failure detection methodology. The implementability and efficiency of the proposed robust control methodology is illustrated through the attitude control of a PVTOL system.

In [17], Escobar *et al.* take the PVTOL model as a simplified unmanned aerial aircraft platform to design new control laws that can be adapted to other vehicles. In this case, they use a PVTOL linearized model through feedback. This linearization does not expand through all the state space, as it presents a singularity. Conditions for local asymptotic stability are determined through a control based on feedback linearization that avoids reaching any singularity. The control design assumes that the speed is small enough that any aerodynamic effects, such as drag, is negligible in the model. To illustrate the effectiveness of the control proposal, numerical simulations are presented. A crosswind perturbation was added; in this scenario the state does not fully reach the desired reference but the error is bounded.

In [18], Lozano *et. al.* propose a modified nested saturation control (inspired in Teel’s paper [19]) to stabilize the model of the PVTOL system. Through numerical simulations, a comparison between this proposal and the one presented in [9] is provided. It is observed that, with the proposed approach, a disturbance in the orientation rate results in a smaller state displacement and the convergence time is shorter. However, the price paid for obtaining these advantages is to reduce the altitude of the PVTOL during a few seconds.

In [20], Sanchez *et. al.* propose a global stabilizer control for PVTOL aircraft using Lyapunov theory and saturation functions. This approach considers that the control inputs are arbitrarily bounded. The proposed solution is tested with real-time experiments. The controller design does not consider the presence of disturbances, however, real-time experiments show that the controller performs well even in the presence of manual perturbations.

In [21], Lozano *et al.* developed a simplified control method to stabilize the PVTOL system with a constant force applied to the horizontal axis, ensuring exponential stability under suitable conditions. The horizontal force is modeled as a spring attached between the system and the environment. The mathematical model considers that there is no coupling between the roll moment and the lateral force. The results of numerical simulations reveal that it is possible to maintain a constant orientation angle by exerting the horizontal force required to elongate the spring a desired distance.

Based on an extended linear state observer and active disturbance rejection control, a novel approach is introduced by Villaseñor Rios *et. al.* in [22] to control a nonlinear underactuated PVTOL aircraft system with an inverted pendular load. The addressed problem consist of designing a control scheme for take-off and landing maneuvers while ensuring the stabilization of the inverted pendulum position around the unstable equilibrium point, in spite of the presence of disturbances caused by crosswind with random amplitudes and unmodeled dynamics. Numerical simulations show the effectiveness of the proposed control scheme. For the sake of comparison, a sliding mode controller derived from a linear approximation of the system was implemented. The chattering problem in sliding mode control does not appear, but the peaking phenomenon arises in the transient response of the sliding mode controller and a larger control amplitude of the proposal is noticed in the transient behavior due to the high gain nature of the controller. The trajectory tracking error in both cases are similar in amplitude, which shows that both controllers are suitable for the task.

Recent results cope with the problem of path tracking of Unmmaned Aerial Vehicles (UAV) in the presence of uncertainties employing sliding mode control along with a variety of techniques such as barrier functions, saturated control (see [23–25]). Sliding mode controllers are commonly implemented to reject bounded uncertain matching perturbations. As explained in [26], a variable structure compensator is comprises two phases. The first one is the reaching phase that uses inverse dynamics to model uncertainties. The second one is the sliding mode, which has discontinuity and uses high control gain to overcome uncertainties and disturbances. A major drawback of the SMC is the chattering phenomena which is a high-frequency switching action which results in high-frequency oscillations of the controlled system [27].

A full review on the control design for a PVTOL aircraft is out of the scope of this study; however, we suggest the work [28], where the interested reader can find a well-developed and structured evaluation of popular control algorithms for the PVTOL.

### 1.1 Contributions

In this study, we are interested in the feedback regulation control problem for a PVTOL aircraft affected by crosswind disturbances. Our approach consists of merging two control techniques, namely:

1. Passivity Lyapunov function control.
2. Integral Sliding Mode (ISM) control.

The first part of the control design is devoted to achieve the regulation of the aircraft to a desired position. To do so, we construct a Lyapunov function which physically represents the kinetic and potential energies of the system. If we locate the rolling angle inside the upper-half plane, the Lyapunov function is positive definite, and proper for any state. Having obtained that function, we straightforwardly obtain the continuous stabilizer controller that ensures asymptotic stability to the desired rest position, within a certain region of guaranteed stability. The analysis makes use of LaSalle’s Invariance Theorem. Notice that the standard passivity control does not cope with disturbances; this is why additional design is required to robustify such control.

The second part of the control aims at cancelling or compensating the matched crosswind disturbances from almost the beginning of the operation. By a suitable selection of the sliding surface which includes the angle-dependent control matrix, we design a unit vector discontinuous control that converges from the initial condition (see [29, 30]). To guarantee the convergence, Lyapunov analysis is carried out, ensuring the expected performance. The advantage of combining both approaches is that it allows working with the nominal system (no disturbance) for the estimation of the attractive region in which the passivity control guarantees stability. Numerical simulations were presented to assess the effectiveness of our control approach.

The rest of the paper is organized as follows. Section 2 introduces the PVTOL aircraft model and establishes the control goal. Section 3 develops the Passivity Lyapunov-based control strategy to solve the PVTOL regulation problem and the complementary discontinuous controller based on the ISM approach. Section 4 presents the numerical experiments designed to assess the effectiveness of our approach and provides a comparative analysis considering two different robust control techniques. Section 5 is devoted to the concluding remarks.

**Notation.** In this manuscript, all vectors and the gradient operator $\frac{\partial }{\partial x}$ are column vectors. The symbol *sgn*(*x*) is used to refer to the sign function of a real number. That is:

$$\begin{array}{c} sgn\left(x\right)=\{\begin{array}{c} 1\;\text{if}\;\;x>0;\\ {}[-1,1]\;\text{if}\;\;x=0;\\ -1\;\text{if}\;\;x<0. \end{array} \end{array}$$

## 2 Dynamical model

**Fig 1:**
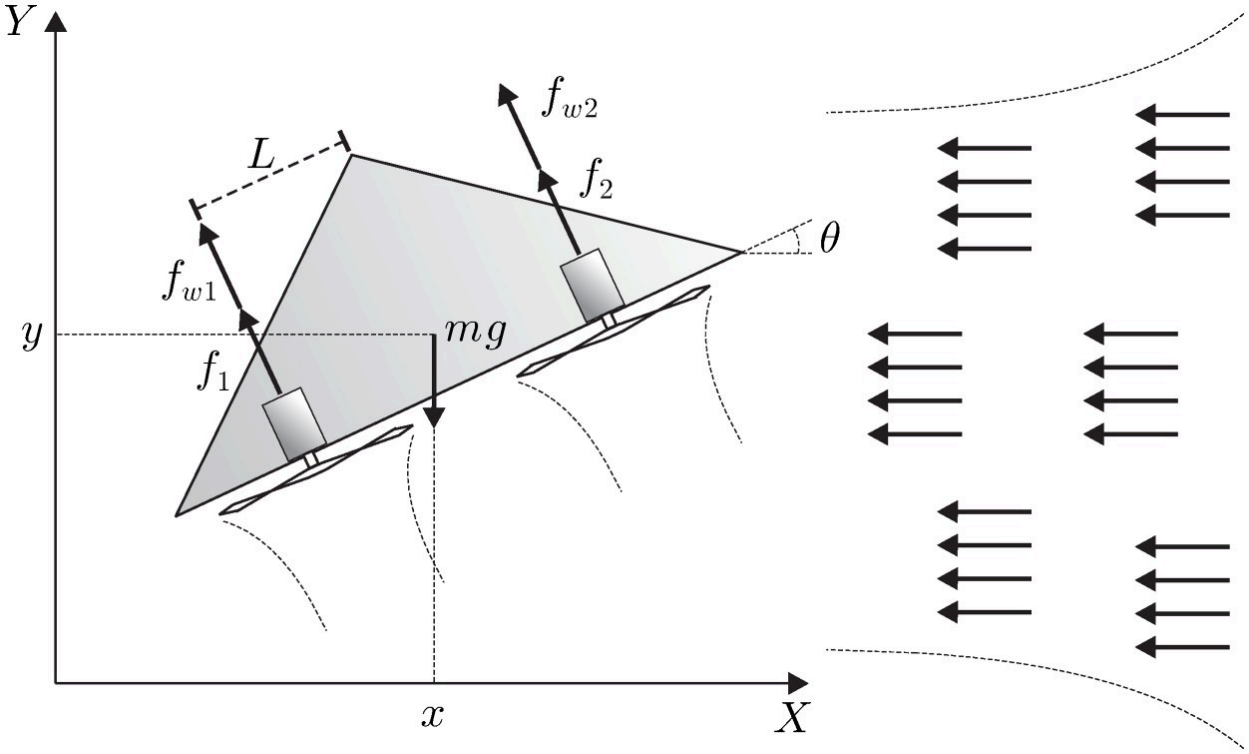
The PVTOL aircraft.

The PVTOL aircraft is an underactuated system with two inputs and three degrees of freedom. The PVTOL moves on a plane and comprises two independent motors that produce a force and a moment on the vehicle (see Fig 1). The following model describes the PVTOL aircraft dynamics [13, 31]:

$$\begin{array}{c} \begin{array}{c} m\overset{..}{X}=-\text{sin}\;\theta \left(f_{1_{T}}+f_{2_{T}}\right)+\varepsilon \;\text{cos}\;\theta \left(f_{1_{T}}-f_{2_{T}}\right)L;\\ m\overset{..}{Y}=\text{cos}\;\theta \left(f_{1_{T}}+f_{2_{T}}\right)+\varepsilon \;\text{sin}\;\theta \left(f_{1_{T}}-f_{2_{T}}\right)L-mg;\\ J\overset{..}{\theta }=\left(f_{1_{T}}-f_{2_{T}}\right)L; \end{array} \end{array}$$ (1)

where *X*, *Y*, and *θ* are the horizontal, vertical, and angular displacements, respectively, *m* is the total aircraft mass, the gravity force acting on the mass center of the aircraft is denoted by *g*, and the moment of inertia is *J*. The length from the mass center of the aircraft to its rotors is *L*. The angle formed by the aircraft and the imaginary horizontal plane is *θ*. The terms $f_{1_{T}}=f_{1}+f_{w_{1}}$ and $f_{2_{T}}=f_{2}+f_{w_{2}}$ represent the forces applied to the aircraft, where *f*_1_ and *f*_2_ are the thrust produced by the rotors, and $f_{w_{1}}$ and $f_{w_{2}}$ correspond to the crosswind forces (a detailed treatment of the crosswind effect, can be found in [13]). The parameter *ε* characterizes the coupling between the rolling moment and the lateral acceleration.

**Remark 1.** *In general, when designing control laws for this type of aircraft, the parameter ε is ignored; however, when performing numerical simulations, it is considered. The latter validates the control law robustness for the nonlinear model. From a practical point of view, ε is not very significant because physically ε < <1. Together with ε, some other elements not considered in practice are difficult to estimate. To overcome this situation, we design a control law robust enough to counteract all the system’s internal nonlinear uncertainties*.

We first normalize the system (1) to facilitate the forthcoming developments. To this end, we use the following changes of variables:

$$\begin{array}{c} \begin{array}{cc} x=\frac{X}{g}; & y=\frac{Y}{g};\\ u_{T_{1}}=\frac{f_{1}+f_{2}}{mg}; & u_{T_{2}}=\frac{(f_{1}-f_{2})L}{J};\\ w_{1}=\frac{f_{w_{1}}+f_{w_{2}}}{mg}; & w_{2}=\frac{(f_{w_{1}}-f_{w_{2}})L}{J}, \end{array} \end{array}$$ (2)

and assuming *ε* = 0, we can rewrite the model (1) as:

$$\begin{array}{c} \begin{array}{c} \ddot{x}=-\text{sin}\;\theta \left(u_{T_{1}}+w_{1}\right);\\ \ddot{y}=\text{cos}\;\theta \left(u_{T_{1}}+w_{1}\right)-1;\\ \ddot{\theta }=u_{T_{2}}+w_{2}. \end{array} \end{array}$$ (3)

In this model, we take into account the considerations of Remark 1, and assume *w*_1_ and *w*_2_ bounded. Now, we introduce the control goal of this study.

**Control Problem.** *The objective consists of proposing two control laws* $u_{T}={\left[u_{T_{1}},u_{T_{2}}\right]}^{T}$ , *to accomplish the regulation problem for the crosswind perturbed PVTOL system defined in* (2), *that is:*

$$\begin{array}{c} \begin{array}{ccc} \underset{t\to \infty }{\text{lim}}∥q-q_{r}∥=0; & & \underset{t\to \infty }{\text{lim}}p=0, \end{array} \end{array}$$

*where q* = (*x*, *y*, *θ*) *and* $p=(\dot{x},\dot{y},\dot{\theta })$, *and q*_*r*_ = (*x*_*r*_, *y*_*r*_, 0)^*T*^ *is the desired final rest position*.

Inside the scope of this study, we introduce the following useful assumptions.

**A1**: The whole system state is measurable.

**A2**: The system is initialized inside of the half-upper plane defined as:

$$\begin{array}{c} D=\{\left(x,y,\theta ,\dot{x},\dot{y},\dot{\theta }\right)\in {ℜ}^{2}\times (-\frac{\pi }{2},\frac{\pi }{2})\times {ℜ}^{3}\}. \end{array}$$

**A3**: The crosswind forces are bounded as $|w_{i}|<\bar{w}$, for *i* = {1, 2}, and $\bar{w}$ is a known. Functions *w*_*i*_ can be discontinuous.

Assumption A1 is restrictive, but we can relax it, as it is done in actual applications, by using a convenient robust observer, as the super-twisting observers proposed in [32–34], the extended-order observers introduced in [35, 36] or the second-order sliding mode observer proposed in [37]. Also, sometimes a combination of a speedometer and a state observer is used to obtain a more accurate velocity measurement. Assumption 2 is considered to ensure that the PVTOL is not initialized with an orientation outside of the interval $\left(-\frac{\pi }{2},\frac{\pi }{2}\right)$ to avoid controllability lost. Finally, Assumption A3 suggests that the thrust force of the motors should be high enough to counteract the crosswind forces’ undesirable instability effect.

### 2.1 Limitations

The proposed solution requires measurement of position and velocity. Besides, parameters *m*, *L*, and *J* have to be known. One advantage of passivity-based control is that it allows us to determine the attraction domain. However, among its drawbacks, the robustness against perturbations is a surprisingly little-studied aspect. The PVTOL aircraft is usually subject to external disturbances such as gusts and crosswinds. To address this problem, in this work, an ISM controller is proposed to compensate for the disturbances from the initial moment. The combination of the passivity control with the ISM approach strengthens the controller.

## 3 Control scheme

As explained above, the task consists in solving the regulation problem under the crosswind effect. To accomplish it, we design a passivity-based control strategy following the ideas introduced in [38, 39]. Afterwards, we incorporate an integral sliding mode-based robust controller. To this end, we first build the candidate Lyapunov function with its respective stabilizing controller without considering the effect of external disturbance *w* = [*w*_1_, *w*_2_]^*T*^. Then, we carry out the corresponding stability analysis based on LaSalle’s Invariance Theorem. Finally, we propose a disturbance rejection controller to eliminate the crosswind effect.

Note that the model (2) can be expressed in a compact form as:

$$\begin{array}{c} \dot{\chi }=A\left(\chi \right)+B\left(\theta \right)\left(u_{T}+w\right) \end{array}$$ (3)

where *χ* = [*q*^*T*^, *p*^*T*^] ∈ *D* ⊂ ℜ^6^ and:

$$\begin{array}{c} \begin{array}{c} A\left(\chi \right)=[\begin{array}{c} p\\ -e_{2} \end{array}];\\ B\left(\theta \right)={\left[\begin{array}{cccccc} 0 & 0 & 0 & -S_{\theta } & C_{\theta } & 0\\ 0 & 0 & 0 & 0 & 0 & 1 \end{array}\right]}^{T}, \end{array} \end{array}$$ (4)

*e*_2_ = [0, 1, 0]^*T*^, and the symbols *S*_*θ*_, *C*_*θ*_ denote sin(*θ*), cos(*θ*), respectively. Notice that *A*(*χ*) ∈ ℜ^6×1^, *B*(*θ*) ∈ ℜ^6×2^, *u*_*T*_, *w* ∈ ℜ^2×1^, and *rank*(*B*(*θ*)) = 2 for all *θ* ∈ ℜ. In the forthcoming developments, we split control *u*_*T*_ as follows:

$$\begin{array}{c} u_{T}=u+u_{I}=[\begin{array}{c} u_{1}\\ u_{2} \end{array}]+[\begin{array}{c} u_{I_{1}}\\ u_{I_{2}} \end{array}], \end{array}$$ (5)

where the passivity-based controller *u*, assuming *w* = 0, is devoted to asymptotically stabilize the PVTOL to the reference position *q*_*r*_, while the integral sliding mode controller *u*_*I*_ compensates the undesirable crosswind effect.

### 3.1 Design of the passivity-based controller *u*

If we consider the case when *w* = 0, we can rewrite the system (2) as:

$$\begin{array}{c} \dot{\chi }=A\left(\chi \right)+B\left(\theta \right)u. \end{array}$$ (6)

The goal is designing a controller *u*(*χ*), such that in closed-loop with the system (6), ensures that:

$$\begin{array}{c} \chi \to \chi_{r}={\left(x_{r},y_{r},0,0,0,0\right)}^{T}. \end{array}$$

To solve the passivity-based problem and change the equilibrium point of (6), we give to this system the structure of the inverted pendulum cart introduced in [38]. To this end, we use the global coordinate variables change, proposed in [39, 40], defined as:

$$\begin{array}{c} \begin{array}{ccc} x_{1}=x-\lambda S_{\theta } & ; & y_{1}=y+\lambda C_{\theta };\\ x_{2}=\dot{x}-\lambda \omega C_{\theta } & ; & y_{2}=\dot{y}-\lambda \omega S_{\theta };\\ & & \omega =\dot{\theta }, \end{array} \end{array}$$ (7)

where λ is a strictly positive control design parameter. It is worth mentioning that the new horizontal and vertical coordinates of the Huygens center of oscillation, *x* and *y*, are, in fact, the system flat outputs, which we can interpret as the dynamics of a pendulum of length λ [5]. Combining (6) and (7), we obtain the following subsystem:

$$\begin{array}{c} \begin{array}{c} {\dot{x}}_{1}=x_{2};\\ {\dot{y}}_{1}=y_{2};\\ \dot{\theta }=\omega ;\\ {\dot{x}}_{2}=-u_{1}S_{\theta }-u_{2}\lambda C_{\theta }+\lambda \omega^{2}S_{\theta }\\ {\dot{y}}_{2}=u_{1}C_{\theta }-u_{2}\lambda S_{\theta }-\lambda \omega^{2}C_{\theta }-1;\\ \dot{\omega }=u_{2}. \end{array} \end{array}$$ (8)

To change the equilibrium point of the above set of equations, we introduce the globally defined input change (suggested in [39, 40]):

$$\begin{array}{c} {}[\begin{array}{c} u_{1}\\ u_{2} \end{array}]={\left[\begin{array}{cc} -S_{\theta } & -\lambda C_{\theta }\\ C_{\theta } & -\lambda S_{\theta } \end{array}\right]}^{-1}[\begin{array}{c} -\lambda \omega^{2}S_{\theta }+\upsilon_{1}\\ 1+\lambda \omega^{2}C_{\theta }+\upsilon_{2} \end{array}], \end{array}$$ (9)

where *υ*_1_ and *υ*_2_ are the new controllers. Substituting the transformation (9) into Eq (8), we obtain:

$$\begin{array}{c} \begin{array}{c} {\dot{z}}_{1}=z_{2};\\ \dot{\theta }=\omega ;\\ {\dot{z}}_{2}=\upsilon ;\\ \dot{\omega }=-\frac{1}{\lambda }S_{\theta }+\frac{1}{\lambda }F^{T}\left(\theta \right)\upsilon , \end{array} \end{array}$$ (10)

where *z*_1_ = [*x*_1_, *y*_1_]^*T*^, *z*_2_ = [*x*_2_, *y*_2_]^*T*^, *υ* = [*υ*_1_, *υ*_2_] and:

$$\begin{array}{c} F^{T}\left(\theta \right)=[-C_{\theta },\;-S_{\theta }]. \end{array}$$ (11)

As we can see, system (10) has a structure similar to the mentioned inverted pendulum cart system.

#### 3.1.1 Total energy and control design

Before designing the control law and proposing the needed Lyapunov function, we introduce the following state variables and a suitable set related to the domain of attraction *D*_*N*_:

$$\begin{array}{c} \begin{array}{cc} q_{N}=[\begin{array}{c} z_{1}\\ \theta \end{array}]; & p_{N}=[\begin{array}{c} z_{2}\\ \omega \end{array}], \end{array} \end{array}$$

with *q*_*N*_ ∈ *D*_*N*_ ∈ ℜ^3^ and *p*_*N*_ ∈ ℜ^3^, and *D*_*N*_ defined as:

$$\begin{array}{c} D_{N}=\{q_{N}\in {ℜ}^{2}\times (-\frac{\pi }{2},\frac{\pi }{2})\}. \end{array}$$ (12)

Then we propose the following energy function:

$$\begin{array}{c} H\left(q_{N},p_{N}\right)=\frac{p_{N}^{T}M\left(\theta \right)p_{N}}{2}+V\left(q_{N}\right), \end{array}$$ (13)

where *M*(*θ*) = *M*^*T*^(*θ*) ∈ ℜ^3×3^ > 0, *V*(*q*_*N*_)>0 for all *q*_*N*_ ∈ *D*_*N*_. Notice that the equilibrium point of (10) is given by $q_{N}=\bar{q}={\left[{\bar{x}}_{1},{\bar{y}}_{1},0\right]}^{T}$. We can say that the equilibrium point is stable in the Lyapunov sense if the function *V*(*q*_*N*_) is positive definite in a neighborhood of $\bar{q}$ with:

$$\begin{array}{c} \bar{q}=\text{arg}\;\text{min}V\left(q_{N}\right), \end{array}$$

and the time derivative of the Lyapunov function *H*(*q*_*N*_, *p*_*N*_) can be expressed as:

$$\begin{array}{c} \dot{H}={\dot{\xi }}^{T}(n\left(q_{N},p_{N}\right)+\alpha \left(\theta \right)\upsilon ). \end{array}$$ (14)

Choosing *υ* as:

$$\begin{array}{c} \upsilon =-\alpha^{-1}\left(\theta \right)(K_{D_{k_{d}}}\dot{\xi }+n), \end{array}$$

with $K_{D_{k_{d}}}>0$, allows ensuring $\dot{H}\leq 0$, where *n* ∈ ℜ^2^ and *α*(*θ*) ∈ ℜ^2×2^ are some suitable functions restricted to *θ* ∈ (−*π*/2, *π*/2), and the auxiliary variable $\dot{\xi }\in {ℜ}^{2}$ is defined below. Besides, if $\dot{\xi }$ is detectable, we can ensure asymptotic stability to the rest position point $\bar{q}$. For detectable, we mean that if $\dot{\xi }=0$, then *p*_*N*_ = 0 and $q_{N}=\bar{q}$ for all *t* > 0.

Having proposed the candidate Lyapunov function, we can design the control strategy. We first select the auxiliary variable $\dot{\xi }\in {ℜ}^{2}$ as [38, 41]:

$$\begin{array}{c} \dot{\xi }=K_{D_{k_{1}}}z_{2}+K_{D_{k_{2}}}\omega F\left(\theta \right), \end{array}$$ (15)

where *S*(*θ*) ∈ ℜ^2^, with $K_{D_{k_{1}}}$ and $K_{D_{k_{2}}}$ are selected as explained below. Now, considering the previously introduced Lyapunov function, we adequate it as:

$$\begin{array}{c} H=\frac{1}{2}{\dot{\xi }}^{T}\dot{\xi }+\frac{k_{p}}{2}{\left(\xi -\bar{\xi }\right)}^{T}(\xi -\bar{\xi })+k_{i}\Phi \left(\theta ,\omega ,z_{2}\right), \end{array}$$ (16)

where *k*_*p*_ > 0, *k*_*i*_ > 0, $\bar{\xi }$ is a suitable constant related to the desired rest position ${\bar{q}}_{N}$, and Φ(*θ*, *ω*, *z*_2_) ∈ ℜ is selected such that the following equation:

$$\begin{array}{c} \omega \frac{\partial \Phi }{\partial \theta }+\dot{\omega }\frac{\partial \Phi }{\partial \omega }+{\dot{z}}_{2}^{T}\frac{\partial \Phi }{\partial z_{2}}=\dot{\Phi }=\upsilon^{T}\dot{\xi }, \end{array}$$ (17)

is fulfilled. Substituting the values of variables $\dot{\omega }$, ${\dot{z}}_{2}$, and $\dot{\xi }$, defined in (10) and (15), in (17) we have:

$$\begin{array}{c} \begin{array}{c} \upsilon^{T}(K_{D_{k_{1}}}z_{2}+K_{D_{k_{2}}}\omega S\left(\theta \right))=\omega \frac{\partial \Phi }{\partial \theta }+(-\frac{1}{\lambda }S_{\theta }+\frac{1}{\lambda }F^{T}\left(\theta \right)\upsilon )\frac{\partial \Phi }{\partial \omega }+\upsilon^{T}\frac{\partial \Phi }{\partial z_{2}} \end{array}. \end{array}$$ (18)

From (18), we easily obtain the following restriction equations:

$$\begin{array}{c} \begin{array}{c} \omega \frac{\partial \Phi (\cdot )}{\partial \theta }-\frac{1}{\lambda }S_{\theta }\frac{\partial \Phi (\cdot )}{\partial \omega }=0;\\ \upsilon^{T}\frac{\partial \Phi (\cdot )}{\partial z_{2}}=\upsilon^{T}K_{D_{k_{1}}}z_{2};\\ \frac{1}{\lambda }\upsilon^{T}F\left(\theta \right)\frac{\partial \Phi (\cdot )}{\partial \omega }=\upsilon^{T}K_{D_{k_{2}}}\omega S\left(\theta \right). \end{array} \end{array}$$ (19)

Evidently, from restrictions (19), a solution for Φ(⋅) is given by:

$$\begin{array}{c} \Phi \left(\theta ,\omega ,z_{2}\right)=K_{D_{k_{2}}}(\left(1-C_{\theta }\right)+\frac{\lambda }{2}\omega^{2})+\frac{K_{D_{k_{1}}}}{2}z_{2}^{T}z_{2}. \end{array}$$ (20)

From (19) and (20), we find that the passivity variable $\dot{\xi }$ is given by:

$$\begin{array}{c} \dot{\xi }=K_{D_{k_{1}}}z_{2}+K_{D_{k_{2}}}\omega F\left(\theta \right), \end{array}$$ (21)

where *F*(*θ*) is given in (11). From (10) and (21), is easy to see that *ξ* is given by:

$$\begin{array}{c} \xi =K_{D_{k_{1}}}z_{1}+K_{D_{k_{2}}}E\left(\theta \right), \end{array}$$ (22)

where *E*(*θ*) is given by:

$$\begin{array}{c} E\left(\theta \right)=\int F\left(\theta \right)\dot{\theta }dt=[\begin{array}{c} -S_{\theta }\\ C_{\theta } \end{array}]. \end{array}$$

Having obtained the auxiliary passivity variable *ξ*, we define the kinetic and potential energies that shape the Lyapunov function. Then, using Eqs (13), (16) and (20), the needed kinetic energy is defined as:

$$\begin{array}{c} \frac{p_{N}^{T}Mp_{N}}{2}=\frac{1}{2}{\dot{\xi }}^{T}\dot{\xi }+\frac{K_{D_{k_{1}}}K_{D_{k_{2}}}\lambda }{2}\omega^{2}, \end{array}$$ (23)

while the potential energy is defined as:

$$\begin{array}{c} V\left(q_{N}\right)=K_{D_{k_{1}}}K_{D_{k_{2}}}\left(1-C_{\theta }\right)+\frac{k_{p}}{2}{\left(\xi -\bar{\xi }\right)}^{T}(\xi -\bar{\xi }), \end{array}$$ (24)

where:

$$\begin{array}{c} \bar{\xi }=K_{D_{k_{1}}}{\bar{z}}_{1}+K_{D_{k_{2}}}E\left(0\right) \end{array}$$

and ${\bar{z}}_{1}={\left[{\bar{x}}_{1},{\bar{y}}_{1}\right]}^{T}$. Note that the design constants have to be positive for the kinetic and potential energies to be positive.

**Remark 2.** *By choosing the set of constants as k*_*p*_ > 0, *k*_*i*_ > 0, $k_{D_{k_{1}}}>0$, *and* $k_{D_{k_{2}}}>0$, *we can ensure that the kinetic energy* (23) *and the potential energy* (24) *are both globally positive definite for all q*_*N*_ ∈ *D*_*N*_, *with the minimum at* ${\bar{q}}_{N}={\left({\bar{x}}_{1},{\bar{y}}_{1},0\right)}^{T}=\left(x_{r},y_{r}+\lambda ,0\right)$ *(see the* S1 Appendix).

Under the conditions of Remark 2, we derive the needed controller. To accomplish it, we obtain the time derivative of *H* defined in (16), with Φ defined in (20). Therefore, computing the time derivative of *H* around the trajectories of the system (10), we obtain the following expression:

$$\begin{array}{c} \dot{H}={\dot{\xi }}^{T}\overset{..}{\xi }+k_{p}{\dot{\xi }}^{T}(\xi -\bar{\xi })+k_{i}\dot{\Phi }. \end{array}$$ (25)

According to Eq (17), we have:

$$\begin{array}{c} \dot{\Phi }={\dot{\xi }}^{T}\upsilon , \end{array}$$ (26)

and computing the second time derivative of *ξ* we obtain:

$$\begin{array}{c} \overset{..}{\xi }=\left(K_{D_{k_{1}}}+\frac{1}{\lambda }K_{D_{k_{2}}}F^{T}F\right)\upsilon -K_{D_{k_{2}}}\omega^{2}E\left(\theta \right)-\frac{1}{\lambda }K_{D_{k_{2}}}S_{\theta }F\left(\theta \right). \end{array}$$ (27)

Substituting the values of (26) and (27) into Eq (25) leads to:

$$\begin{array}{c} \dot{H}={\dot{\xi }}^{T}(\eta \left(q_{N,}p_{N}\right)+\alpha \left(\theta \right)\upsilon ), \end{array}$$ (28)

where:

$$\begin{array}{c} \begin{array}{c} n\left(q_{N,}p_{N}\right)=-K_{D_{k_{2}}}\omega^{2}E\left(\theta \right)-\frac{1}{\lambda }K_{D_{k_{2}}}S_{\theta }F\left(\theta \right)+k_{p}(\xi -\bar{\xi });\\ \alpha \left(\theta \right)=k_{i}+K_{D_{k_{1}}}+\frac{1}{\lambda }K_{D_{k_{2}}}F\left(\theta \right)F^{T}\left(\theta \right). \end{array} \end{array}$$ (29)

Based on Eq (28), it is convenient to select the stabilizing controller as:

$$\begin{array}{c} \upsilon =\alpha^{-1}\left(\theta \right)(-K_{D_{k_{d}}}\dot{\xi }-n\left(q_{N,}p_{N}\right)), \end{array}$$ (30)

with *k*_*d*_ strictly positive constant, ensuring that:

$$\begin{array}{c} \dot{H}=-{\dot{\xi }}^{T}K_{D_{k_{d}}}\dot{\xi }. \end{array}$$

Notice that any initial condition (*q*_*N*_(0), *p*_*N*_(0)) with |*θ*| < *π*/2 satisfying:

$$\begin{array}{c} H\left(q_{N}\left(0\right),p_{N}\left(0\right)\right)<c=k_{i}K_{D_{k_{2}}}, \end{array}$$

ensures *H*(*q*_*N*_(*t*), *p*_*N*_(*t*)) < *c* for all *t* > 0, restricted to |*θ*| < *π*/2, since *H* is a strictly positive definite and a non-increasing function. That is, we can define an invariant set as:

$$\begin{array}{c} \Omega_{c}=\{\left(q_{N}^{T},p_{N}^{T}\right)\in D_{N}\times {ℜ}^{3}:H\left(q_{N},p_{N}\right)\leq \underline{c}\}, \end{array}$$ (31)

where *D*_*N*_ is defined in (12), $\underline{c}=k_{i}K_{D_{k_{2}}}-\delta$, with 0 < *δ* sufficiently small. In other words, Ω_*c*_ defines an invariant set related to the attraction domain. In the following section, we develop the corresponding convergence proof.

### 3.2 Asymptotic stability

Having shaped the candidate Lyapunov function *H*, with its stabilizing controller, we show that the obtained closed-loop system is globally asymptotically stable for any initial condition (*q*_*N*_(0), *p*_*N*_(0)) starting inside of the invariant set Ω_*c*_.

Let us begin by introducing the following helpful proposition.

**Proposition 1.** *Consider system* (10) *in closed-loop with:*

$$\begin{array}{c} \upsilon =\alpha^{-1}\left(\theta \right)(-K_{D_{k_{d}}}\dot{\xi }-n\left(q_{N,}p_{N}\right)), \end{array}$$ (32)

*where α* and *n are defined in* (29), $\dot{\xi }$ *is defined in* (21), *and the design constants are defined as in Remark 2. Then, for any initial condition* (*q*_*N*_(0), *p*_*N*_(0)) ∈ Ω_*c*_, *the rest position* $\bar{q}=\left({\bar{x}}_{1},{\bar{y}}_{1},0\right)$ *is an asymptotically stable equilibrium point, with domain of attraction defined in* (12).

**Proof.** To prove the stability of the system (10) in closed-loop with the controller (32), we consider the previously constructed Lyapunov function (25), positive definite and radially unbounded for all (*q*_*N*_, *p*_*N*_) ∈ Ω_*c*_, the time derivative of *H* around the trajectories of the closed-loop system, and the already proved inequality:

$$\begin{array}{c} \dot{H}\left(q_{N},p_{N}\right)=-{\dot{\xi }}^{T}K_{D_{k_{d}}}\dot{\xi }\leq 0, \end{array}$$

where $K_{D_{k_{d}}}=K_{D_{k_{d}}}^{T}>0$ holds. Then, we conclude that (*q*_*N*_, *p*_*N*_) are bounded and stable in the Lyapunov sense. Even more, because $\dot{H}\leq 0$, for any initial condition starting in Ω_*c*_, we also have that (*q*_*N*_, *p*_*N*_) remain in Ω_*c*_. To ensure asymptotic convergence, we apply the Invariance Theorem of LaSalle. To this end, we define the invariant set Γ as:

$$\begin{array}{c} \Gamma =\{\left(q_{N}\left(t\right),p_{N}\left(t\right)\right)\in \Omega_{c}:{\dot{\xi }}^{T}K_{D_{k_{d}}}\dot{\xi }=0\}, \end{array}$$ (33)

and let *N* be the largest invariant set in Γ. To compute the set *N* ⊂ Γ, we note that $\dot{\xi }=0$ in the set Γ. Therefore, $\overset{..}{\xi }=0$ and *ξ* = ξ_*_, where ξ_*_ is a constant. From Eqs (25) and (28), and the definition of the controller (30), we can easily see that the latter was designed fulfilling the following equation:

$$\begin{array}{c} \overset{..}{\xi }+k_{p}(\xi -\bar{\xi })+k_{i}\upsilon =-K_{D_{k_{d}}}\dot{\xi }. \end{array}$$ (34)

Substituting the corresponding values of *ξ*, $\dot{\xi }$, and $\overset{..}{\xi }$ in the set Γ, we obtain the following equation:

$$\begin{array}{c} k_{p}(\xi_{*}-\bar{\xi })+k_{i}\upsilon =0. \end{array}$$

From the above, we conclude that the controller *υ* in the set Γ is a constant defined by:

$$\begin{array}{c} \upsilon =\frac{-k_{p}\left(\xi_{*}-\bar{\xi }\right)}{k_{i}}=\bar{\upsilon }. \end{array}$$ (35)

From Eq (35), we analyze two possibilities:

1) According to Eq (10), if $\bar{\upsilon }=0$ and $\xi_{*}=\bar{\xi }$, then ${\dot{z}}_{2}=0$ and $z_{2}={\bar{z}}_{2}$, with ${\bar{z}}_{2}$ constant. But, if ${\bar{z}}_{2}\neq 0$ in the set Γ, then ‖*z*_1_(*t*)‖ is not bounded on Γ. This fact leads to a contradiction since (*q*_*N*_(*t*), *p*_*N*_(*t*)) ∈ Ω_*c*_. Hence, $\{z_{1}={\bar{z}}_{1}$, $z_{2}=0,\upsilon =0,\xi =\bar{\xi }=\xi_{*}\}$ in the set Γ and, from the definition of $\dot{\xi }$ in (21), we conclude that *ω* = 0 and $\theta =\bar{\theta }$, with $\bar{\theta }$ constant belonging to (−*π*/2, *π*/2). Substituting these values in the second time derivative of *ξ* given in (27), we have the following relation:
  $$\begin{array}{c} 0=\text{sin}\bar{\theta }[\begin{array}{c} \text{cos}\bar{\theta }\\ -\text{sin}\bar{\theta } \end{array}], \end{array}$$
  implying that $\bar{\theta }=0$, because $|\bar{\theta }|<\pi /2$. Consequently, *θ* = 0 in the set Γ. That is, $q_{N}=\bar{q}$ and *p*_*N*_ = 0 in the set Γ.
2) Complementary, if $\bar{\upsilon }\neq 0$ in the set Γ, then, once again from Eq (10), ‖*z*_1_‖ and ‖*z*_2_‖, that is the norms of the states, are unbounded, which is a contradiction, because $q_{N}=\left(z_{1}^{T},\theta \right)$ and $p_{N}=\left(z_{2}^{T},\omega \right)$ are bounded in the set Γ. This fact leads us to the case 1. That is, once again $\left\{q_{N}=\bar{q},p_{N}=0\right\}$ in the set Γ. Therefore, the maximal invariant set *N* ⊂ Γ is constituted by the single invariant point $N=\{\left(q_{N}=\bar{q},p_{N}=0\right)\}\subset \Gamma$. Consequently, from the Invariance Theorem of LaSalle, we have that all solutions starting in Ω_*c*_ ⊂ ℜ^6^, asymptotically converge to the largest invariant set *N*.

Based on the proof of Proposition 1, we introduce the following proposition that ensures the stability of the original system (6).

**Proposition 2.** *Consider system* (6) *in closed-loop with* (9), *where the auxiliary input υ* = [*υ*_1_, *υ*_2_]^*T*^ *is obtained from Proposition 1 in combination with the suitable transformations* (7). *Then, the whole state and our control law ensure asymptotically stability of the desired equilibrium point χ* = (*x*_*r*_, *y*_*r*_, 0, *p*^*T*^ = 0), *with almost all the upper-half plane as its domain of attraction*.

Since this proposition is a direct consequence of Proposition 1 and the suitable transformations, we omit its proof.

#### 3.2.1 A version of the proposed control to simplify the computation of *υ*

To simplify the derivation of the controller *υ*, we can use a filter to estimate $\overset{..}{\xi }$ from the knowledge of $\dot{\xi }$. To this end, we propose the following tracking differentiator:

$$\begin{array}{c} \begin{array}{c} \dot{\xi_{1}}=\xi_{2};\\ \dot{\xi_{2}}=-R^{2}(K_{D_{w1}}\left(\xi_{1}-\dot{\xi }\right)+\frac{1}{R}K_{D_{w2}}\xi_{2});\\ \overset{..}{\hat{\xi }}=\xi_{2}, \end{array} \end{array}$$ (36)

where *w* > 0 and *R* > >1 (see [42]). If we assume that $\overset{...}{\xi }$ is bounded and *R* is sufficiently large, then $\xi_{2}\to \overset{..}{\xi }$ in a very short time. Then, according to Eq (25), we can use the following:

$$\begin{array}{c} \dot{H}=\dot{\xi }\left(\xi_{2}+k_{p}(\xi -\bar{\xi })+k_{i}\upsilon \right). \end{array}$$ (37)

Therefore, the control *υ* can be substituted by:

$$\begin{array}{c} \upsilon =\frac{1}{k_{i}}(-K_{D_{k_{d}}}\dot{\xi }-k_{p}(\xi -\bar{\xi })-\xi_{2}). \end{array}$$ (38)

Notice that the previous controller version *υ* can be seen as a PID controller.

### 3.3 Active compensation of matching perturbation

To improve the robustness of the control law (5), we use the integral sliding mode control approach which guarantees disturbance rejection from the initial time instant [43]. The complementary discontinuous controller *u*_*I*_ is devoted to counteract the undesirable crosswind effect *w* considered in the normalized and compact model (3). For the design of this controller, we propose the suitable sliding manifold {*χ*|*s*(*χ*, *t*) = 0}, defined as:

$$\begin{array}{c} s\left(\chi ,t\right)=B^{T}\left(\theta \right)\Delta_{\chi }\left(t,t_{0}\right), \end{array}$$ (39)

where

$$\begin{array}{c} \Delta_{\chi }\left(t,t_{0}\right)=\chi \left(t\right)-\chi \left(t_{0}\right)-\int_{t_{0}}^{t}(A(\chi (\tau ))+B(\theta (\tau ))u(\tau ))d\tau , \end{array}$$

with *A* and *B* previously defined in (4). Notice that *s*(*χ*, *t*) ∈ ℜ^2^ and *rank B*(*θ*) = 2. Then, computing the time derivative of (39), we obtain:

$$\begin{array}{c} \dot{s}\left(\chi ,t\right)=\dot{\theta }\frac{dB^{T}\left(\theta \right)}{d\theta }\Delta_{\chi }\left(t,t_{0}\right)+B^{T}(\dot{\chi }-A\left(\chi \right)-B\left(\theta \right)u). \end{array}$$

Substituting the value of $\dot{\chi }$ given in (3), considering that *B*^*T*^(*θ*)*B*(*θ*) = *I*_2_, and using the definition in (5), we obtain:

$$\begin{array}{c} \dot{s}\left(\chi ,t\right)=u_{I}+w+\dot{\theta }\frac{dB^{T}\left(\theta \right)}{d\theta }\Delta_{\chi }\left(t,t_{0}\right). \end{array}$$ (40)

From the above, we propose the discontinuous controller as:

$$\begin{array}{c} u_{I}=-\lambda \frac{s}{∥s∥}-\dot{\theta }\frac{dB^{T}\left(\theta \right)}{d\theta }\Delta_{\chi }, \end{array}$$ (41)

where λ > 0 sufficiently large Moreover, the closed-loop system (40) and (41) is given by:

$$\begin{array}{c} \dot{s}=-\lambda \frac{s}{∥s∥}+w. \end{array}$$ (42)

Notice that the gain λ guarantees the enforcement of the state motion on the sliding manifold. Ideally, in an actual environment, the ISM controller would keep zero the difference between the nominal system and the perturbed evolutions if *rank B*(*θ*) = 6. However, in our case, *rank B*(*θ*) = 2, and the perturbation *w* is in the *span B*(*θ*), which means we can reject the perturbation altogether. Then, to carry out the stability analysis, we use the following Lyapunov function:

$$\begin{array}{c} V=\frac{1}{2}s^{T}\left(\chi \right)s\left(\chi \right). \end{array}$$

Computing the time derivative of *V* around the trajectories of (42), we have after some simple manipulations the following:

$$\begin{array}{c} \dot{V}\leq -∥s∥(\lambda -\bar{w})<0,\text{for}\;\text{all}\;s\neq 0. \end{array}$$

Consequently, we can ensure that *s* → 0 in finite time if the following condition is matched:

$$\begin{array}{c} \lambda >\bar{w},\;\;\;\forall t\geq t_{0}. \end{array}$$

On the other hand, because *s*(0) = 0 the following holds:

$$\begin{array}{c} V\left(s(0)\right)=\frac{1}{2}{∥s(0)∥}^{2}=0, \end{array}$$

implying that there is no reaching phase.

**Remark 3.** *The proposed control* (41) *becomes (on average) the estimation of the cumulative term appearing in* (42). *We can see it using the equivalent control technique. In fact, using* (41), *the equivalent control is:*

$$\begin{array}{c} u_{eq}=-(w+\dot{\theta }\frac{dB^{T}\left(\theta \right)}{d\theta }\Delta_{\chi }\left(t,t_{0}\right)), \end{array}$$

*which can be recovered by filtering the control* (41), *with a low pass filter (see* [44]). *In short, the discontinuous control* (41) *compensates the matched uncertain term from the initial time because in some way is an estimation of it. We can do that because the control and the uncertainty are in the same subspace (the span of matrix B*). *Achieving such compensation from the initial time guarantees that the nominal control can work to stabilize the system states (see* [29, 45–47]).

**Fig 2:**
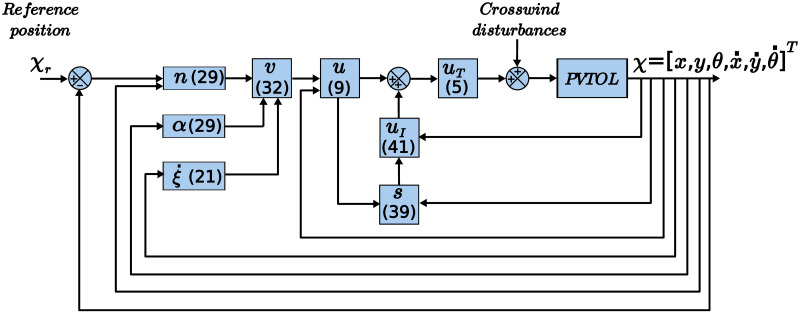
Block diagram of the control scheme.

Fig 2 shows a schematic of the control proposal.

## 4 Numerical simulations

The performance of the proposed control approach is highlighted through four numerical examples:

1. The reference signal corresponds to a pulse train-shaped trajectory. We represent the crosswind effect through sinusoidal functions.
2. The reference signal corresponds to an ellipse-shaped trajectory. We represent the crosswind effect through random, uniformly distributed signals.
3. The task consists of rendering the PVTOL from an initial to a rest position, considering unmatched perturbations.
4. A comparison of our control solution with respect to two other well-established robust approaches when performing a regulation task.

Numerical simulations were conducted using the software Matlab R2014a using a workstation equipped with an Intel(R) Core(TM) i5–4440 CPU @ 3.10GHz processor and an Intel(R) HD 4600 graphics card featuring 2GB of video memory. The system had 8 GB of RAM memory.

Since there is no rule to estimate the control gains using the passivity Lyapunov approach, we tuned them by trial and error. We considered the following gain values:

**Table:** 

*k*_*p*_	$K_{D_{k_{d}}}$	$K_{D_{k_{1}}}$	$K_{D_{k_{2}}}$	*k*_*i*_	λ
1.21	2.2	2	1.5	5	0.7

For the PVTOL, we set its mass as *m* = 0.433*kg*, the distance between its rotors is *L* = 0.163*m*, and we fixed the moment of inertia as *J* = 0.0552*kgm*^2^.

**Example 1.** In this example, the PVTOL is required to track a pulse train-shaped trajectory, defined as:

$$\begin{array}{c} \begin{array}{c} x_{r}=\rho \left(t\right)g;\\ y_{r}=\left(\rho \left(t\right)/2+1.5\right)g;\\ \rho \left(t\right)=sgn(\text{sin}\left(\frac{t}{10}\right)); \end{array} \end{array}$$

considering the initial condition:

$$\begin{array}{c} \begin{array}{ccc} x_{0}=1.96m; & \theta_{0}=0.5rad; & y_{0}=1.96m;\\ {\dot{x}}_{0}=0; & {\dot{\theta }}_{0}=0; & {\dot{y}}_{0}=0 \end{array} \end{array}$$

To simulate the normalized crosswind effect, we use the following functions:

$$\begin{array}{c} \begin{array}{c} w_{1}=\frac{f_{w_{1}}+f_{w_{2}}}{mg}=0.25+0.25{\text{sin}}^{2}\left(\frac{t}{4}\right);\\ w_{2}=\frac{L(f_{w_{1}}-f_{w_{2}})}{Jg}=0.25+0.2\text{cos}\left(\frac{t}{4}\right)\text{sin}\left(\frac{t}{4}\right). \end{array} \end{array}$$

**Fig 3:**
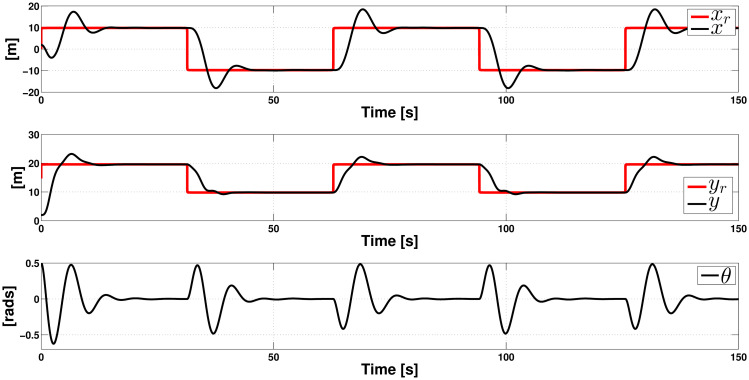
Closed-loop response in the presence of crosswind when tracking a pulse train (position variables).

**Fig 4:**
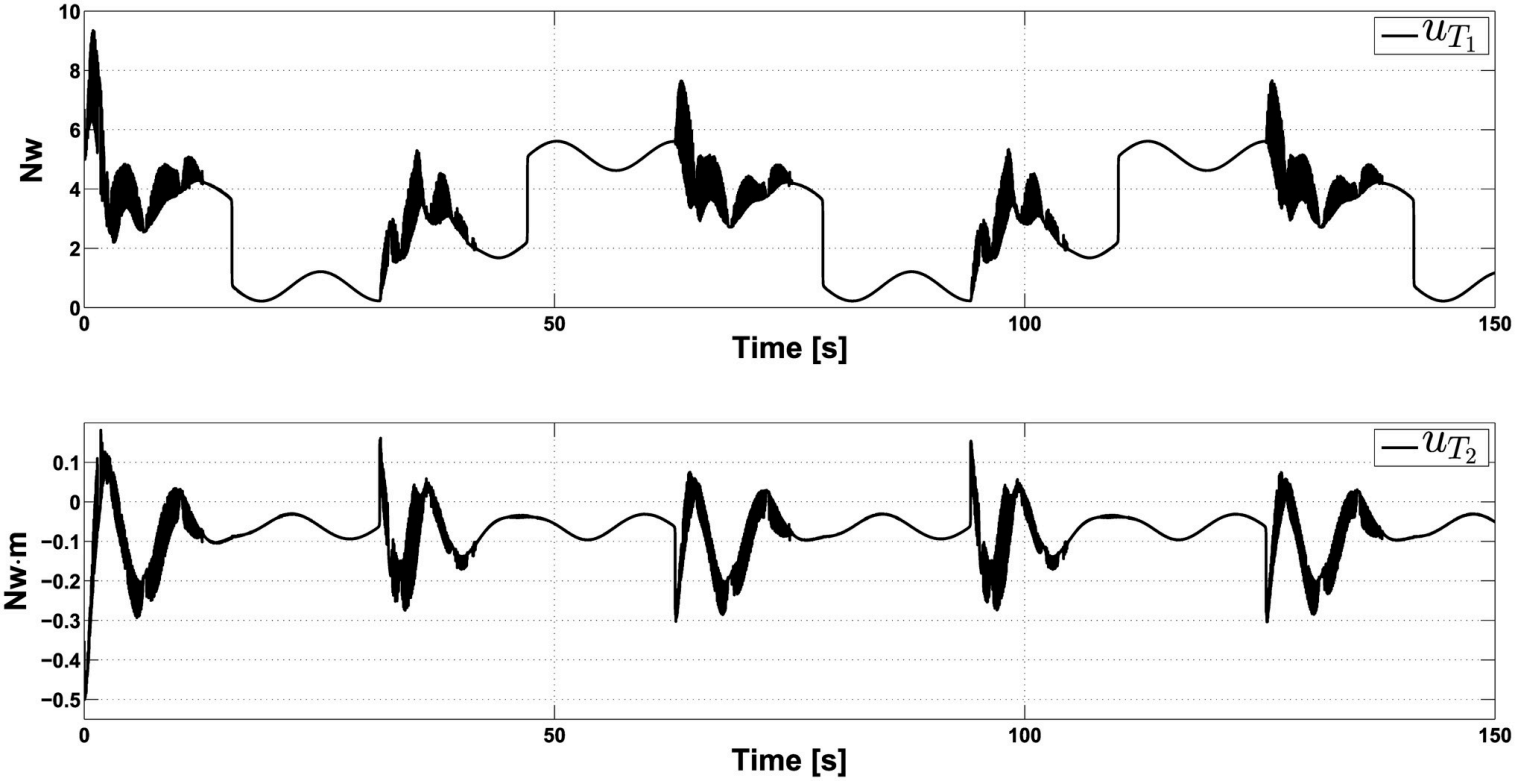
Controllers $u_{T_{1}}$ and $u_{T_{2}}$ behavior in the presence of crosswind, when tracking a pulse train.

We show the experiment outcomes in Figs 3 and 4. One can note that our control approach performs considerably well. Fig 3 shows that the position coordinates *x* and *y* follow the pulse train-shaped trajectory very close. As evident, we can see the pulse train as a trajectory with abrupt variations when its sign changes. That explains why the coordinates *x* and *y* converge to the pulse train, on average, after 17 seconds every time it changes its sign. Also, this figure shows that the angle *θ* converges to the equilibrium point, except in the discontinuities of function *ρ*(*t*). Notice that the performance of the passivity-based control is not affected by the disturbances; the ISM control is able to reject them from the initial time, as expected. Finally, Fig 4 shows the control actions of $u_{T_{1}}$ and $u_{T_{2}}$. Note that the controllers self-adjust to track the pulse train-shaped trajectory and counteract the destabilizing effects of the matching perturbations.

**Example 2.** In this experiment, we tested the performance and behavior of our control approach on a task for which it was not designed. The experiment consists of tracking an ellipse-shaped trajectory. To challenge the proposed controller, a random uniformly distributed signal *r*_*i*_(*t*) ∈ [−0.5, 0.5], (*i* = 1, 2) is considered to represent the external crosswind disturbances. As mentioned, this experiment is of interest because, even when the controller was designed to accomplish regulation, it can track the target trajectories *q*_*r*_ = (*x*_*r*_, *y*_*r*_, 0)^*T*^, such that $∥{\dot{q}}_{r}∥≃0$. The control task consists in tracking the ellipse-shaped reference, defined by:

$$\begin{array}{c} \begin{array}{cc} x_{r}=\text{sin}\left(\frac{t}{15}\right); & y_{r}=1+0.5\;\text{cos}\left(\frac{t}{15}\right), \end{array} \end{array}$$

where the crosswind that externally perturbs the system was fixed as:

$$\begin{array}{c} \begin{array}{ccc} w_{1}^{\prime }=r_{1}\left(t\right)+w_{1}\left(t\right); & & w_{2}^{\prime }=r_{2}\left(t\right)+w_{2}. \end{array} \end{array}$$

**Fig 5:**
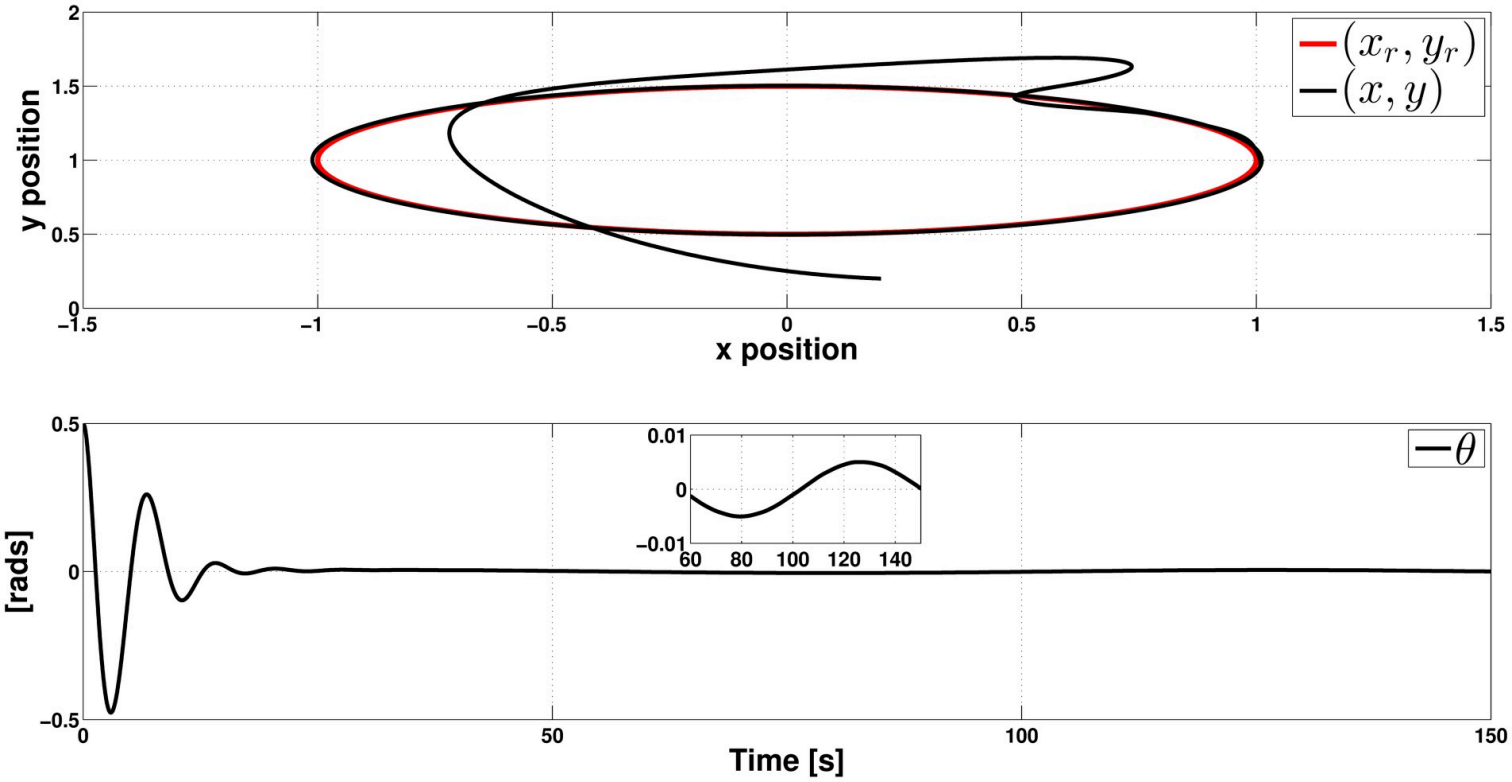
Closed-loop response in the presence of crosswind for an ellipse-shaped reference-tracking task, when the reference is perturbed by the random uniformly distributed signal *r*_*i*_(*t*) (position variables).

**Fig 6:**
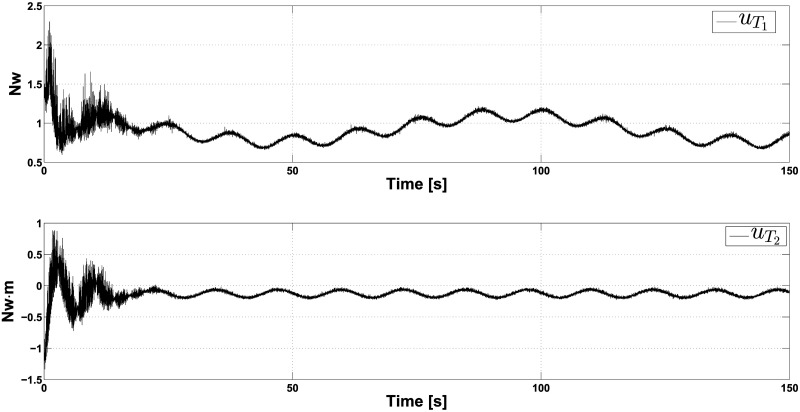
Controllers $u_{T_{1}}$ and $u_{T_{2}}$ behavior in the presence of crosswind for an ellipse-shaped reference-tracking task, when the reference is perturbed by the random uniformly distributed signal *r*_*i*_(*t*).

In Figs 5 and 6, the outcome of this simulation is presented. The top of Fig 5 shows that the controllers effectively make the PVTOL approximately track the ellipse-shaped trajectory. The behavior shown in this figure confirms that the ISM controller can immediately counteract (again from the initial time) the randomly uniformly distributed and bounded perturbation undesirable effect. The bottom of Fig 5 shows the evolution of *θ*, which converges very close to zero after 25 seconds and, from this moment, reaches a steady state. Fig 6 shows the control actions $u_{T_{1}}$ and $u_{T_{2}}$. We can see that both controllers exhibit abrupt variations. After 25 seconds, they reach a steady state, even when we see chattering phenomena due to the undesirable effect of signal *r*_*i*_(*t*). Finally, in Fig 6, we see the tracking errors of positions *x* and *y*. The corresponding steady-state tracking error in both cases is ±0.15*m*.

**Example 3.** In this experiment, the robustness of the controller is tested through the task of driving the system from the first experiment initial position *q*_0_ = (1.96*m*, 1.96*m*, 0.5*rad*), to the final rest position *q*_*r*_ = (9.8*m*, 29.06*m*, 0*rad*), under the crosswind perturbation considered in Example 1. During the experiment, we consider unmatched perturbations in *x* and *y* directions defined as:

$$\begin{array}{c} \begin{array}{c} r_{x}\left(t\right)=0.21\left(\text{sin}\left(t/2\right)\text{cos}\left(t/2\right)\right);\\ r_{y}\left(t\right)=0.42\left(\text{sin}\left(t/2\right)\text{cos}\left(t/2\right)\right). \end{array} \end{array}$$

**Fig 7:**
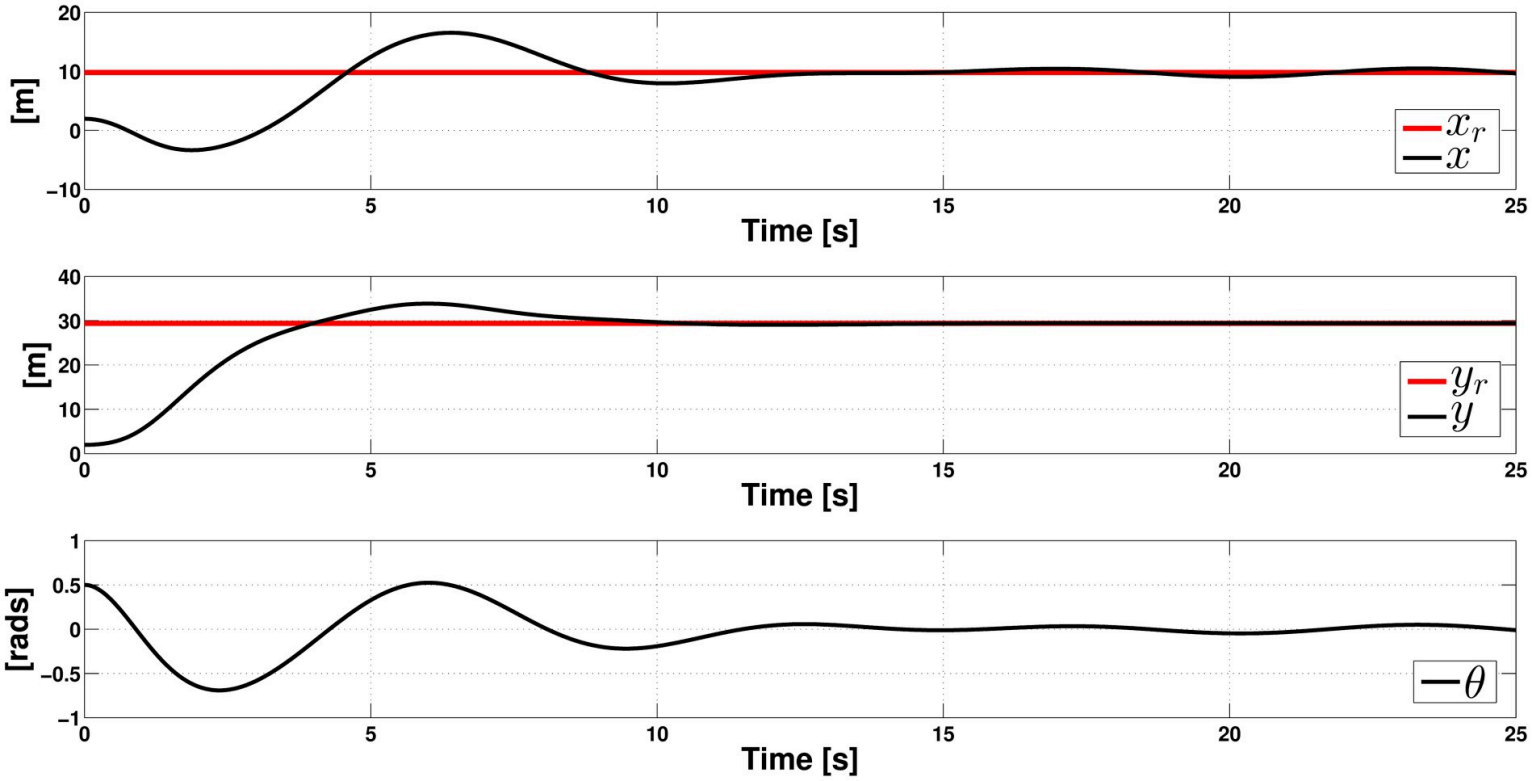
Closed-loop response in the presence of crosswind and unmatched perturbations (position variables).

**Fig 8:**
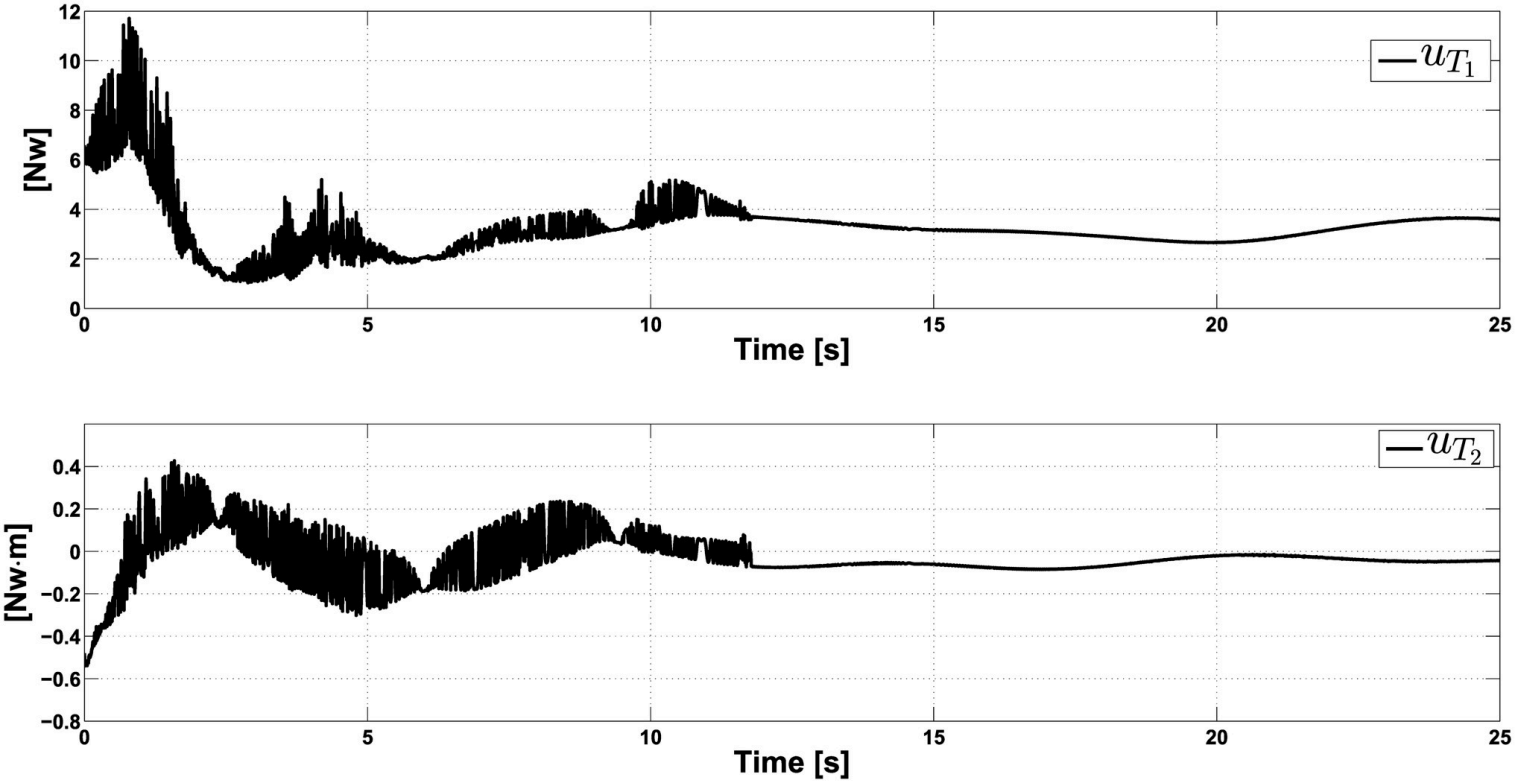
Controllers $u_{T_{1}}$ and $u_{T_{2}}$ behavior in the presence of crosswind and unmatched perturbations.

The obtained results are shown in Figs 7 and 8. In Fig 7, we can see that after 15 seconds, the controller can render the system very close to the final rest position, even when we did not design it to counteract unmatched perturbations. In Fig 8, we can see that the controller $u_{T_{1}}$ is unable to counteract the effect of the unmatched perturbation *r*_*x*_ in the coordinate *x*, as it does in the coordinate *y*, where the undesirable effect is completely compensated and reaches its steady-state after 15 seconds. Regarding the controllers, we can see that they exert considerable effort at the beginning of the operation, reaching their steady state after 12 seconds. In conclusion, we can say that the ISM controller can counteract the undesirable effect of unmatched perturbation on coordinate *y*.

**Example 4.** Numerical comparison between our solution and two well-established robust control approaches to accomplish a regulation task:

1. The control strategy based on Robust Control Lyapunov Functions (RCLF) and Sontag’s universal stabilizing feedback proposed in [13].
2. The output feedback sliding mode controller introduced in [37].

In what follows, our controller will be denoted by O while the ones presented by Muñoz *et. al* in [13] and by Cardenas *et. al* in [37] will be referred to as M and C, respectively.

We select the following piece-wise trajectory:

$$\begin{array}{c} \begin{array}{cc} x_{r}=9.8m; & y_{r}=\{\begin{array}{c} 14.7m,\forall [0,30]s,\\ 24.5m,\forall (30,60]s. \end{array}. \end{array} \end{array}$$

We fixed the initial condition equal to zero, except for the angle, which we set as *θ*(0) = 0.25*rads*. The control parameters for M and C are the ones used in the corresponding references, and the parameters for O are the ones we used in the two previous experiments.

**Fig 9:**
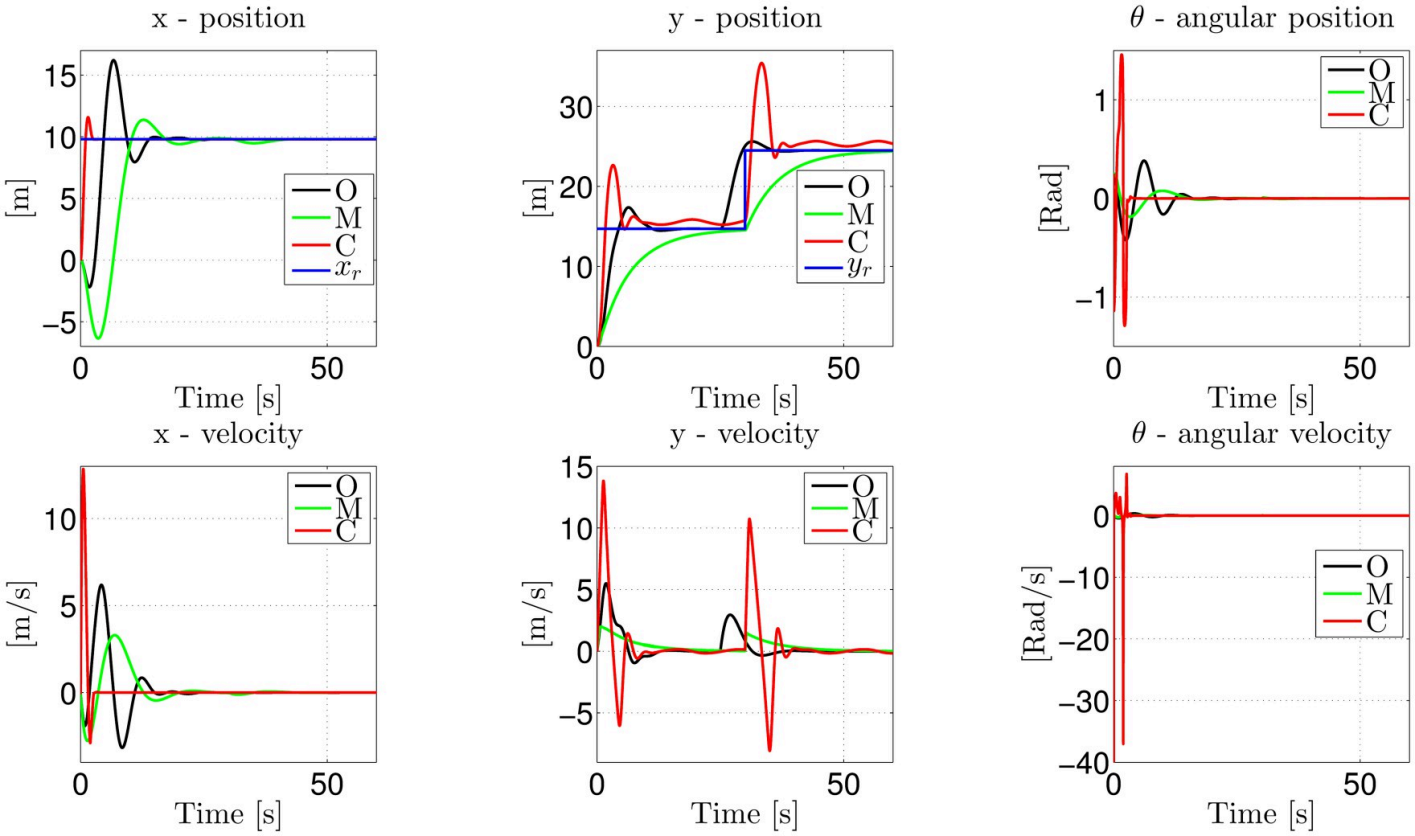
Comparison of the closed-loop response of O, M, and C, when performing a piece-wise reference trajectory.

We show the outcome of this comparison in Fig 9, where we can see that in the *x*-axis, the three strategies have good behavior; in fact, they reach the reference at approximately 12 seconds. On the other hand, over the *y*-axis, we can see that strategies O and C respond better to reference abrupt changes in the *y*-axis. Strategy M has the worst behavior; it takes more time to reach the reference after the abrupt change at 30 seconds. On the other hand, O outperforms M and C in the angular position, with C being the one with the worst performance.

Regarding the velocities, strategy O has smoother behavior than the other two. Once again, strategy C has the worst performance. We must note that we do not include the controller plots because they are based on variable structure, making them very difficult to interpret.

**Fig 10:**
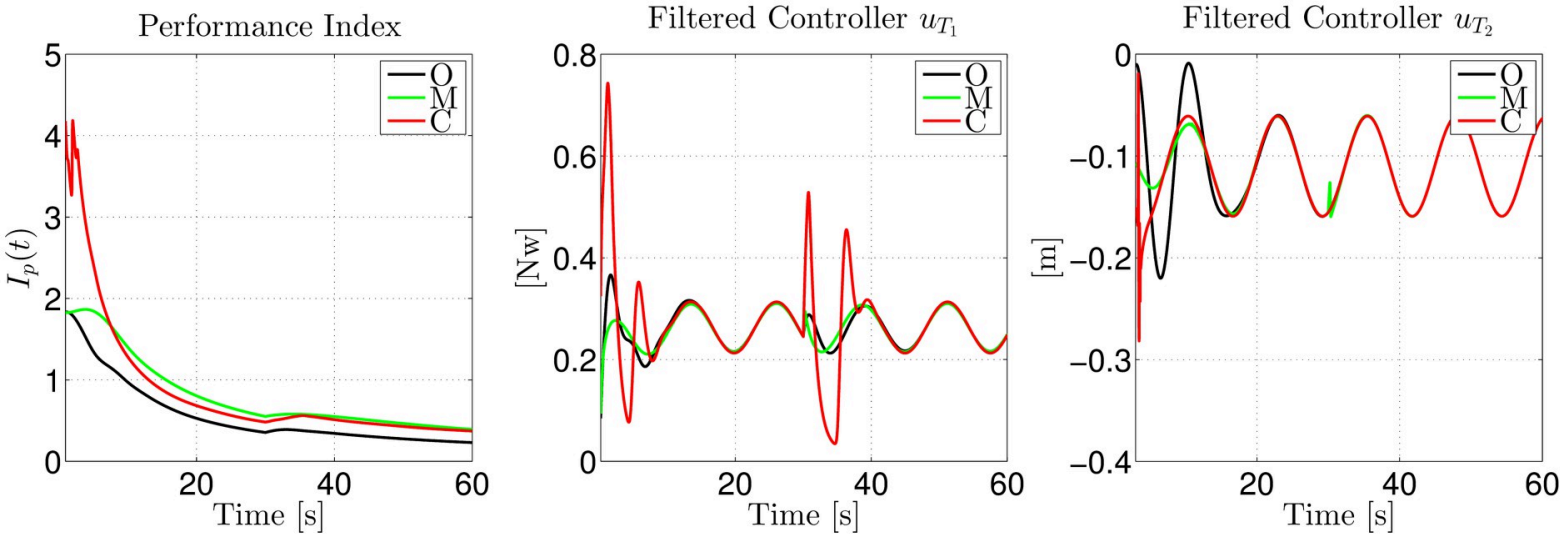
Comparison of the performance indexes and the filtered controllers’ responses for $u_{T_{1}}$ and $u_{T_{2}}$ when performing a piece-wise reference trajectory.

Finally, in the first plot of Fig 10, we show the performance index of control strategies O, M, and C, while the filtered signals of controllers $u_{T_{1}}$ and $u_{T_{2}}$ are shown in the second and third plots, respectively. This figure shows that the three approaches, O, M, and C, have comparable performance indexes, with O having a slightly better performance. Related to the filtered controllers shown in the second and third plots, we can see that all three also have similar behavior in the steady state. For instance, we can see that the three filtered controllers for $u_{T_{1}}$ overlap during the interval [15, 30]*s*. Then, due to the abrupt change in reference *y*_*r*_ at 30 seconds, there is a discrepancy among the three controllers’ responses, which again overlap at 42 seconds. We can see a similar behavior for the three filtered controllers for $u_{T_{2}}$.

Recall that the performance index of a signal *χ*, with 0 ≤ *t* ≤ *T*, is defined as:

$$\begin{array}{c} I_{P}\left(T\right)=\frac{1}{T}\int_{0}^{T}∥\chi \left(s\right)-\chi_{r}∥ds, \end{array}$$

and the filtered control signals are given by:

$$\begin{array}{c} {\tilde{u}}_{i}\left(s\right)=\frac{u_{T_{i}}\left(s\right)}{s+1};i=\left\{1,2\right\} \end{array}$$

### 4.1 Some considerations about our control proposal implementation

a) Unfortunately, to our knowledge, there is no general rule or procedure for tuning the control parameters when the controller is passivity-based for the PVTOL particular case. In our case, we empirically tuned the gains such that the controller exhibited acceptable performance—a short response time, avoiding the picking phenomenon. The controller parameters have been selected in order to reduce the convergence time of the error between the PVTOL system’s position and its desired value.
b) Migrating our control solution to an actual aircraft can be successfully done, provided we know the system’s actual parameter values, have accurate aircraft position measurements, and equip the aircraft with precise speedometers for the position and angular velocities. On the other hand, having accurate filtered signals is, maybe, the main obstacle to our approach. We can overcome this drawback through a convenient Kalman filter implementation, together with an Inertial Measure Unit (IMU) or a video recording camera [13, 48]. Future work of this paper will consider experiments in an ad hoc platform. A basic setup for an experimental PVTOL includes the following items:
  1. Manufacturing of the PVTOL vehicle, there are several options for materials that can be wood, plastic, 3D print, and design trust in a plane, etc. (see [1, 2]).
  2. Propels (pitch angle, length), motors (RPM, Voltage) and
  3. ESCs (electronic speed controllers), and IMU (inertial measure unit)
  4. Hardware for data acquisition can be aboard the A/D converters.
  5. Software for programming the control of the motors and communication with sensors.
  6. Sensor for knowing the position of the vehicle.

## 5 Conclusions

We solved a robust feedback regulation control law for a simplified PVTOL aircraft model under the crosswind effect based on a Lyapunov passivity-based control combined with the ISM approach. Our control approach comprises two controllers: a continuous one to stabilize the aircraft to a desired rest position and a discontinuous one to counteract the disturbances actively. The surface design of such integral control guarantees the cancellation of the undesirable disturbance effect from the initial time as long as the perturbation bounds are known. To accomplish it, we first shape a Lyapunov function, which physically represents the kinetic and potential energies of the system. The obtained Lyapunov function is positive definite and proper for any state if we locate the rolling angle inside the upper-half plane. Having proposed the candidate Lyapunov function, we obtained the stabilizing controller straightforwardly. We carried out the stability analysis of the feedback control law by applying the Invariance Theorem of LaSalle. To test the effectiveness of our control approach, we designed and ran three numerical simulations, having obtained convincing results. In future work, to avoid the perturbation’s bound knowledge necessity, we can explore a different slide mode-based technique for the perturbation fast identification or use an additional disturbance observer, like the ones proposed by the Active Disturbance Rejection Control.

## Supporting information

S1 Appendix Positiveness of matrix *M*. (DOCX)

S1 File (TEX)
