# Supplementary material for: Robust regulation of a PVTOL aircraft subject to crosswind disturbances: Passivity and integral sliding mode approach
Source: PLoS One. 2024 Dec 5;19(12):e0307398. doi: 10.1371/journal.pone.0307398 (PMC11620607; doi:10.1371/journal.pone.0307398)
Supplement: S1 Appendix — (DOCX) [file pone.0307398.s001.docx]

**Supporting information**

**Appendix. Positiveness of matrix** $M$

From Equation (23), it is easy to see that the kinetic energy can be written as:

$$p_{N}^{T}Mp_{N}=p_{N}^{T}\left[ \begin{matrix} k_{D_{k_{1}}}^{2} & 0 & {-k}_{D_{k_{1}}}k_{D_{k_{2}}}C_{\theta} \\ 0 & k_{D_{k_{1}}}^{2} & {-k}_{D_{k_{1}}}k_{D_{k_{2}}}S_{\theta} \\ {-k}_{D_{k_{1}}}k_{D_{k_{2}}}C_{\theta} & {-k}_{D_{k_{1}}}k_{D_{k_{2}}}S_{\theta} & k_{D_{k_{2}}}^{2}+k_{D_{k_{1}}}k_{D_{k_{2}}}\lambda\end{matrix} \right]p_{N,}$$

where $M>0$, $\det\left( M \right)=k_{D_{k_{1}}}^{5}k_{D_{k_{2}}}\lambda>0$. On the other hand, the potential energy $V(q)$,

defined in (24) is, evidently, positive definite for $q_{N}\in\left\{ \mathfrak{R}^{2}\times\left( -\pi/2,\pi/2 \right) \right\}$, $k_{p}>0$, $k_{D_{k_{1}}}>0,$and $k_{D_{k_{2}}}>0$, with minimum at $q_{N}=\left( \bar{x},\bar{y},0 \right).$
